# OP50 BRISA: Regional Database Of Health Technology Assessment Reports Of The Americas

**DOI:** 10.1017/S0266462325101086

**Published:** 2025-12-29

**Authors:** Malen Hollmann, Vania Cristina Canuto Santos, Flavia Ribeiro Salomon, Caroline Bregman, Alexandre Lemgruber

## Abstract

**Introduction:**

The Regional Database of Health Technology Assessment Reports of the Americas (BRISA) provides free access to bibliographic information on health technology assessment (HTA) prepared and published by member institutions of the HTA Network of the Americas (RedETSA) and strategic partners. BRISA is the first open regional virtual library that centralizes HTA reports which would otherwise be scattered or inaccessible to the public.

**Methods:**

BRISA was developed in collaboration with the Latin American and Caribbean Center on Health Sciences Information (BIREME) through the Virtual Health Library. The platform and the methodology for registering, organizing, and disseminating reports were developed by BIREME, in coordination with the Pan American Health Organization. HTA reports are shared in Spanish, Portuguese, French, and English. A retrospective analysis of BRISA’s development and achievements since its launch was conducted. The analysis included the number of countries and institutions providing HTA reports, the number of HTA reports published over time, the number of users per country, and the total number of page views since its creation.

**Results:**

Since its launch in 2017, the number of contributors to BRISA has reached 14 countries and more than 40 institutions. BRISA is constantly growing and in 2024, it reached more than 4,000 reports. In 2024, Brazil, Mexico, and Argentina had the highest number of users (with 16,620, 12,750, and 9,338 users, respectively). The number of page views increased from 4,833 in 2018 to more than 840,000 in 2024. Since 2024, BRISA also includes HTA reports from partner networks like the Spanish Network of Agencies for HTA and Services of the National Health System (RedETS) in Spain.

**Conclusions:**

BRISA is a collaborative network that facilitates learning from the experiences of other countries, helps avoid duplication of effort, and promotes the incorporation of cost-effective health technologies and their appropriate use. With the BRISA database, RedETSA seeks to promote the use of HTA to improve the decision-making process for incorporating technologies in health systems, improving access and equity.

